# Population susceptibility to a variant swine-origin influenza virus A(H3N2) in Vietnam, 2011–2012

**DOI:** 10.1017/S0950268815000187

**Published:** 2015-03-12

**Authors:** L. N. M. HOA, J. E. BRYANT, M. CHOISY, L. A. NGUYET, N. T. BAO, N. H. TRANG, N. T. K. CHUC, T. K. TOAN, T. SAITO, N. TAKEMAE, P. HORBY, H. WERTHEIM, A. FOX

**Affiliations:** 1 Oxford University Clinical Research Unit and Wellcome Trust Major Overseas Programme, Vietnam; 2 Nuffield Department of Medicine, Oxford University, Oxford, UK; 3 MIVEGEC (UMR Universités Montpellier 1 & 2, CNRS 5290, IRD 224), Montpellier, France; 4 Hanoi Medical University, Hanoi, Vietnam; 5 Influenza and Prion Diseases Research Center, National Institute of Animal Health, National Agriculture and Food Research Organization (NARO), Ibaraki, Japan; 6 The University of Melbourne, Peter Doherty Institute for Infection and Immunity, Department of Microbiology and Immunology, Parkville, Victoria, Australia

**Keywords:** Influenza A, virology (human) and epidemiology, zoonoses

## Abstract

A reassortant swine-origin A(H3N2) virus (A/swine/BinhDuong/03-9/2010) was detected through swine surveillance programmes in southern Vietnam in 2010. This virus contains haemagglutinin and neuraminidase genes from a human A(H3N2) virus circulating around 2004–2006, and the internal genes from triple-reassortant swine influenza A viruses (IAVs). To assess population susceptibility to this virus we measured haemagglutination inhibiting (HI) titres to A/swine/BinhDuong/03-9/2010 and to seasonal A/Perth/16/2009 for 947 sera collected from urban and rural Vietnamese people during 2011–2012. Seroprevalence (HI ⩾ 40) was high and similar for both viruses, with 62·6% [95% confidence interval (CI) 59·4–65·7] against A/Perth/16/2009 and 54·6% (95% CI 51·4–57·8%) against A/swine/BinhDuong/03-9/2010, and no significant differences between urban and rural participants. Children aged <5 years lacked antibodies to the swine origin H3 virus despite high seroprevalence for A/Perth/16/2009. These results reveal vulnerability to infection to this contemporary swine IAV in children aged <5 years; however, cross-reactive immunity in adults would likely limit epidemic emergence potential.

Studies of the human–swine interface for influenza A virus (IAV) transmission are important to understand the complex multi-host ecology of influenza, and to assess potential disease risks. IAVs currently circulating in global swine populations comprise three subtypes (H1N1, H1N2, H3N2), and are similar to those circulating in humans (H1N1 and H3N2). Recent large-scale assessments of IAV diversity in swine have highlighted the frequency of human-to-swine transmission (so-called ‘reverse zoonoses’), which appears to be far more common than swine-to-human transmission [1]. In particular, human H3N2 viruses have entered swine populations on numerous occasions, beginning in 1980s in Europe, in the 1990s in North America, and later observed in many countries and all continents [1–4]. Typically, onward transmission of the human-origin viruses in swine has led to subsequent reassortment, and divergence into lineages with mixed genome constellations of swine, human, and avian origin [2]. Importantly, the process of antigenic drift that is characteristic of H3N2 evolution in human populations is believed to operate on a slower time scale in swine [1]. Thus, swine IAV with envelope haemagglutinin (HA) and neuraminidase (NA) proteins derived from ‘old’ human seasonal strains – i.e. viruses that have since disappeared from human circulation – may continue to circulate in swine for many years with relatively few antigenic changes, and disease risks associated with these variant viruses remain poorly understood.

In recent years, surveillance of swine influenza viruses in Vietnam has received much attention, due to the perception of Vietnam as a ‘hotspot’ for viral emergence and the inherent dangers of co-circulation of highly pathogenic avian viruses (H5N1) in areas with dense poultry and pig populations. The first detection of human-origin influenza viruses in swine in Vietnam was a reassortant A(H3N2) (A/swine/BinhDuong/03–9/2010, and hereafter referred to as Sw/VN10) found in 2010 from a commercial pig farm located in the southeast region of Vietnam, to the north of Ho Chi Minh City [5]. Genetic analysis revealed that the six Binh Duong H3N2 swine viruses isolated at one time from the same farm had HA similar to human seasonal viruses circulating between 2004 and 2006 (Fig. 1) [5]. The estimated time to most recent common ancestor of the Vietnamese swine-origin Binh Duong H3 cluster suggested human-to-pig transmission in late 2004 (95% highest posterior density value, 23 October 2004 to 16 December 2004). Antigenically, Sw/VN10 exhibited a profile that was distinguishable from contemporary human H3 viruses, but showed some level of cross-reactivity with antisera of human seasonal vaccines and recent cluster variants (Table 1, e.g. 1:320 to A/Wyoming/03 and 1:140 to A/Wuhan/95). Although swine surveillance data from Vietnam is not available prior to 2010, Sw/VN10-like viruses were isolated each year from southern Vietnamese swine from 2010 to 2014, suggesting that it is has been one of the dominant contemporary swine IAVs circulating in Vietnam, at least for the past 4 years. The human seasonal H3N2 viruses circulating in the region during 2007–2008 were A/H3N2/Brisbane/10/2007-like, with a cluster transition to A/Perth/16/2009 (hereafter referred to as Pe09) in 2009 that has persisted until at least mid-2013.

**Fig. 1.**
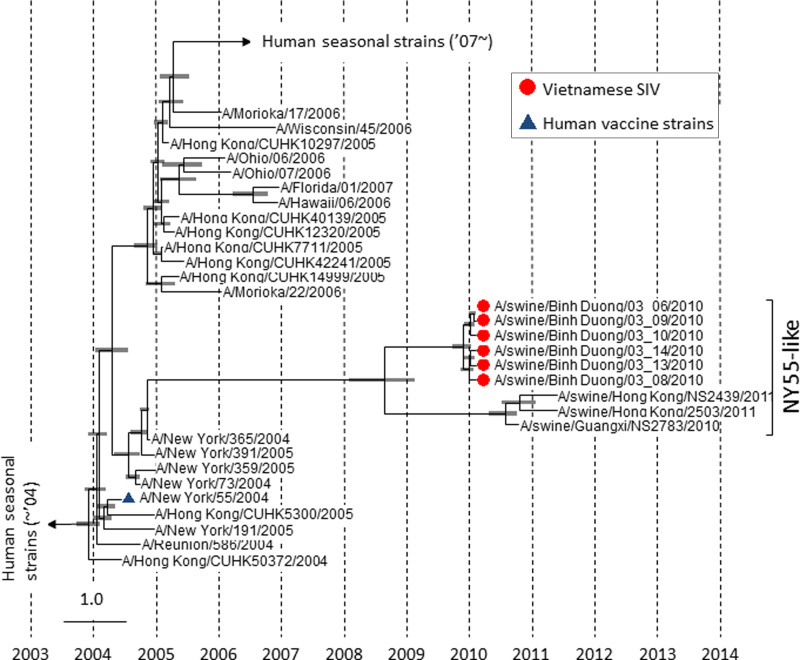
Bayesian Markov chain Monte Carlo phylogenetic tree based on nucleotide sequences of H3 HA genes of selected H3N2 viruses. The analysis was performed in Beast package v. 1.7.4 (http://beast.bio.ed.ac.uk/) using 164 HA sequences (1701 bp) of H3N2 viruses isolated from 2000 to 2013. Node bars show the estimated divergent period to most recent common ancestor (95% highest posterior density).

**Table 1.** Antigenic characterization of Sw/VN10 by haemagglutination inhibition using reference chicken antisera to Vietnamese swine and avian H3 viruses, and ferret antisera to human H3N2 vaccine strains

Virus	HI titres with							
	Hyperimmune chicken antisera against							
	Vietnamese swine IAV	AIV	Infected ferret sera					
			Human vaccine strains					
	BD014/10	Bud77	Wuh95	Syd97	Wyo03	NY04	Uru07	Vic11
Human-like SIVs								
A/swine/Binh Duong/03-9/2010 (H3N2)	1280	<40	160	40	320	40	<40	<40
A/swine/Binh Duong/03-14/2010 (H3N2)	**2560**	<40	160	40	320	80	40	40
Seasonal human virus								
A/Port Chalmer/1/1973 (H3N2)	<40	<40	<40	<40	<40	<40	<40	<40
A/Victoria/3/1975 (H3N2)	<40	<40	<40	<40	<40	<40	<40	<40
A/Bangkok/1/1979 (H3N2)	80	<40	<40	40	40	40	<40	<40
A/Wuhan/359/1995 (H3N2)	<40	<40	**320**	40	40	40	<40	<40
A/Sydney/5/1997 (H3N2)	<40	<40	640	**1280**	640	40	<40	<40
A/Wyoming/3/2003 (H3N2)	40	<40	320	320	**2560**	2560	1280	<40
A/New York/55/2004 (H3N2)	<40	<40	<40	<40	1280	**5120**	1280	<40
A/Uruguay/716/2007 (H3N2)	<40	<40	<40	<40	160	1280	**2560**	40
A/Perth/16/2009 (H3N2)	<40	<40	<40	<40	<40	40	80	320
A/Victoria/361/2011 (H3N2)	<40	<40	<40	<40	80	160	320	**1280**
Avian influenza virus								
A/budgerigar/Aichi/1/1977 (H3N8)	<40	**10240**	<40	<40	<40	<40	<40	<40

IAV, Influenza A virus; AIV, avian influenza virus; SIV, swine influenza virus.

Homologous titres are indicated in bold.

In this study, we aimed to investigate the susceptibility of the Vietnamese human population to Sw/VN10 compared to contemporary circulating human H3N2 (Pe09) using serum collections from a previous comparative study of urban and rural residential populations [6]. A total of 943 participants were enrolled: 495 urban residents from Hanoi city and 448 rural residents of Ba Vi district, located 60 km southwest of Hanoi. About 40% of participants were aged <20 years (mean age 33 years, range 2–91 years); sampling was age-stratified and designed to over-represent children aged <20 years, hence the age distribution of samples did not reflect population structure. A single serum sample was obtained from each of the rural participants (between March and May 2012), whereas paired serum collections were obtained from the urban participants (278 pairs, collected in October 2011 and February/March 2012). Prior to performing HI analysis, the reactivity of the two antigens using guinea pig and turkey erythrocytes was evaluated in parallel. Both the Pe09 and Sw/VN10 antigens exhibited twofold higher HA titres when using turkey *vs*. guinea pig, and the agglutination phenotype was considerably more stable using turkey erythrocytes. Hence, HI assays were performed using standard methods with 0·5% turkey erythrocytes and a starting dilution of 1:10. For single sera analysis, ‘seropositive’ was defined as a titre of ⩾40; for paired sera analysis, ‘seroconversion’ was defined as a fourfold or greater rise in HI titre, with a second titre of at least 40.

The overall seroprevalence of titres ⩾40 against Pe09 and Sw/VN10 was 62·6% [95% confidence interval (CI) 59·4–65·7] and 54·6% (95% CI 51·4–57·8), respectively (Table 2). Correlation analysis of Pe09 and Sw/VN10 HI titres by Spearman coefficient was 0·426 (*r*_*s*_ = 82316028, *P* < 0·001), consistent with significant cross-reactivity to the two antigens. In children aged <5 years, only 9/70 (12·9%) were seropositive to Sw/VN10, whereas 48/70 (68·6%) were seropositive to Pe09; these proportions were highly significantly different (*χ*^2^ = 42·7, d.f. = 1, *P* < 0·001). Figure 2 shows the polynomial logistic regressions of seroprevalence as a function of age for Pe09 and Sw/VN10. Titres to Pe09 were highest in children aged <15 years, whereas highest titres to Sw/VN10 were in young adults aged 15–25 years, with very low titres observed in children aged <10 years, and a trough in titres for people aged 40–60 years. We propose that this pattern of higher reactivity in young adults reflects the influenza strains that individuals may have first experienced as young children, i.e. A/Wuhan/95 and A/Wyoming/03. Sex ratios were not significantly different between the two sites (*χ*^2^ = 0·0105, d.f. = 1, *P* = 0·9182). However, mean ages were higher in Dong Da (34·8 years) than in Ba Vi (31·2 years) (*t* = 2·335, d.f. = 936·809, *P* = 0·020). Despite this, the age profile of HI titres was not different between the two sites, neither for Pe09 (likelihood ratio test: *χ*^2^ = 0·202, d.f. = 1, *P* = 0·6533), nor for Sw/VN10 (likelihood ratio test: *χ*^2^ = 1·266, d.f. = 1, *P* = 0·2606) (Supplementary Fig. S1).

**Fig. 2.**
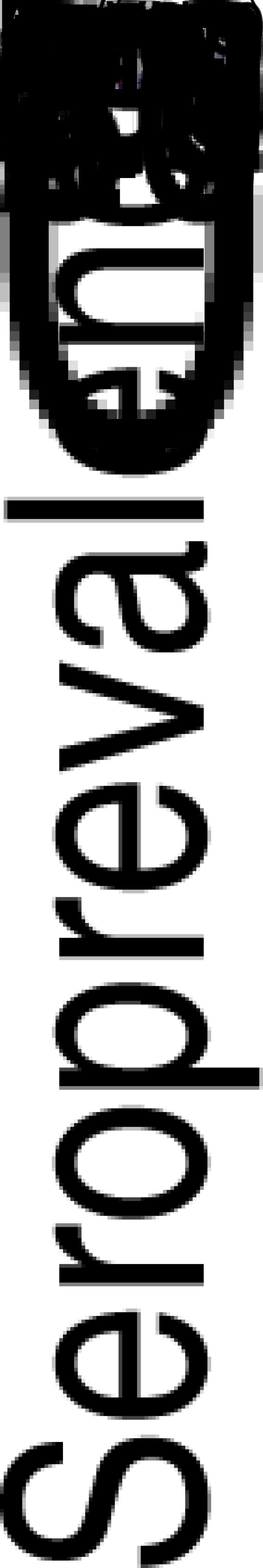
Percentage of individuals with an HI titre ⩾40 (%) against Pe09 (blue) and Sw/VN10 (red). Dots and vertical bars show the mean seroprevalences and their 95% confidence intervals for the nine age groups defined by the thin vertical grey lines. The limits of these age groups were chosen so that they all contain approximately the same number of samples. The curves show the models of the polynomial logistic regressions, up until degree 5 (degree 6 being non-significantly different from 0). The coloured area shows the 95% confidence intervals of the models' predictions.

**Table 2.** Demographic characteristics of the study participants and haemagglutinin inhibition (HI) antibody titres by age and influenza strain. Seropositive defined as titre ⩾40; seroconversion defined by fourfold or greater rise in HI titre, with a second titre at least 1:40

Age group, yr	Total no. sera	No. pair sera	Mean age, yr	Female sex (%)	No. Dong Da (urban)	No. Ba Vi (rural)	Seropositives (%)		GMT (95% CI)		Seroconversions (%)	
							A/H3/Pe09	A/Sw/VN10	A/H3/Pe09	A/Sw/VN10	A/H3/Pe09	A/Sw/VN10
<5	70	13	3·7	47·1	31	39	48 (68·6%)	9 (12·8%)	5·8 (5·3–6·2)	3·2 (2·9–3·4)	1 (7·7%)	0 (0%)
5–9	121	31	7·5	45·5	51	70	103 (85·1%)	52 (43%)	6·2 (5·9–6·5)	4·5 (4·2–4·8)	2 (6·4%)	1 (3·2%)
10–14	126	32	12·3	52·4	62	64	98 (77·8%)	105 (83·3%)	5·7 (5·4–5·9)	6·2 (5·9–6·4)	4 (12·5%)	1 (3·1%)
15–19	83	29	17·6	54·2	53	30	53 (63·9%)	72 (86·7%)	5·3 (4·9–5·6)	6·6 (6·2–7·0)	1 (3·4%)	1 (3·4%)
20–29	83	19	25·4	55·4	48	35	52 (62·7%)	62 (74·7%)	5·1 (4·8–5·5)	6·1 (5·7–6·4)	1 (5·3%)	0 (0%)
30–39	114	28	35·2	64	60	54	60 (52·6%)	61 (53·5%)	4·7 (4·5–5·0)	4·8 (4·6–5·1)	3 (10·7%)	4 (14·3%)
40–49	86	24	44·7	56·9	37	49	41 (47·7%)	31 (36%)	4·5 (4·2–4·8)	4·3 (3·9–4·6)	2 (8·3%)	0 (0%)
50–59	93	30	55·1	63·4	51	42	48 (51·6%)	41 (44·1%)	4·6 (4·4–4·9)	4·5 (4·2–4·8)	3 (10%)	1 (3·3%)
60–69	84	39	65·4	48·8	51	33	42 (50%)	41 (48%)	4·6 (4·3–4·9)	4·8 (4·4–5·2)	5 (12·8%)	3 (7·7%)
>69	83	33	77·2	55·4	51	32	45 (54·2%)	41 (49·4%)	4·9 (4·5–5·3)	4·9 (4·4–5·3)	9 (27·2%)	3 (10%)
Overall	943	278	33·1	54·4	495	448	590 (62·6%)	515 (54·6%)	5·2 (5·1–5·3)	5·0 (4·9–5·2)	31 (11·1%)	14 (5·03%)

GMT, Geometric mean titre of log_2_-transformed HI titre; CI, confidence interval.

Analysis of paired sera from 278 urban participants found 11·1% (31/278) with seroconversions to Pe09 and 5·0% (14/278) with seroconversions to Sw/VN10, of whom five were dual seroconversions to both Sw/WN10 and Pe09. Dual seroconverters were exclusively found in the 30–39 and >69 years age groups (Supplementary Table S1). Influenza vaccination status of participants indicated that only 51 had been vaccinated against influenza within the past year, 842 had not been vaccinated within the past year, and 43 did not know their status. In people that reported recent vaccination (for which the H3 vaccine virus would have been Pe09), HI titres against Pe09 were higher compared to those who were not vaccinated, and there were no seroconversions in vaccinated people compared to 11·4% (95% CI 9·4–13·8) seroconversions to Pe09 in non-vaccinated individuals (Supplementary Fig. S2). Titres to Sw/VN10 were similar in both vaccinated and non-vaccinated individuals [7, 8].

In summary, we found that more than 50% of the Vietnamese population had HI titres ⩾40 to Sw/VN10; however, the variable age profile of HI reactivity suggested significant differences in susceptibility to infection, with the greatest vulnerability in children aged <5 years. In previous behavioural surveys conducted within the same populations, rural inhabitants of Ba Vi reported significantly more contact with swine than urban residents (40% of rural residents reported direct contact with pigs on ‘most days’ whereas 95% of urban residents ‘never’ had direct contact with pigs) (Supplementary Fig. S3). Given that both rural and urban cohorts exhibited similar overall prevalence to the two antigens, and given the distinct age profiles of immunity, it seems highly unlikely that the observed titres to Sw/VN10 reflect exposure to swine viruses. Rather, we infer that the profile of reactivity to Sw/VN10 observed in adults is consistent with their previous exposures to seasonal H3 strains that were antigenically similar to Sw/VN10. This is compatible with previous studies showing persistent titres to older antigenic clusters [9, 10], and also underscores the need for comprehensive testing of antibody response profiles across multiple antigenic clusters when studying zoonotic risks. Similarly, the relatively low seroprevalence of children aged <5 years to Sw/VN10 most likely reflects lack of infection with ‘old’ human seasonal H3 variants, as well as lack of infection with Sw/VN10-like swine IAVs.

Our findings highlight the susceptibility of children to infection with reassortant swine-human viruses whose HA antigens are divergent from human H3 viruses circulating during their lifetimes. With increasing information about regional and global swine IAV circulation, and more swine IAV isolates being generated by national surveillance projects, it may become feasible to use population immunity data and new computational approaches to predict which swine IAVs pose the greatest threat of zoonotic emergence. However, our findings reveal the significant challenge of using seroepidemiological approaches to assess swine-to-human transmission events, and the need for more comprehensive assessments of population immunity profiles to multiple current and past H3 viruses of human and swine origin. Given that multiple different IAVs with swine-human hybrid genomes continue to circulate in pig populations, the epidemiological relevance of persistent ‘old’ (‘extinct’) human influenza virus variants in swine populations merits further research.

## Supplementary material

For supplementary material accompanying this paper visit https://doi.org/10.1017/S0950268815000187. click here to view supplementary material
