# Supplementary material for: Population susceptibility to a variant swine-origin influenza virus A(H3N2) in Vietnam, 2011–2012
Source: Epidemiol Infect. 2015 Mar 12;143(14):2959–64. doi: 10.1017/S0950268815000187 (PMC4595856; doi:10.1017/S0950268815000187)
Supplement: Supplementary file 1 [file S0950268815000187sup001.doc]

**SUPPLEMENTARY MATERIAL**

**Supplementary Table S1**.Seroconversions to Pe09 and Sw/VN10 by age group.

| **Age group** | **Total No. paired sera** | **A/H3/Pe09 only** | **A/Sw/VN10 only** | **Dual seroconversion** |
| --- | --- | --- | --- | --- |
| **<5** | 13 | 1 | 0 | 0 |
| **5-9** | 31 | 2 | 1 | 0 |
| **10-14** | 32 | 4 | 1 | 0 |
| **15-19** | 29 | 1 | 1 | 0 |
| **20-29** | 19 | 1 | 0 | 0 |
| **30-39** | 28 | 1 | 2 | 2 |
| **40-49** | 24 | 2 | 0 | 0 |
| **50-59** | 30 | 3 | 1 | 0 |
| **60-69** | 39 | 5 | 3 | 0 |
| **>69** | 33 | 6 | 0 | 3 |
| **Total** | **278** | **26** | **9** | **5** |

**Supplementary Figure S1**. Age profile of HI log_2_ titers to Pe09 (blue) and Sw/VN10 (green) stratified by urban (Dong Da) and rural (BaVi) site.


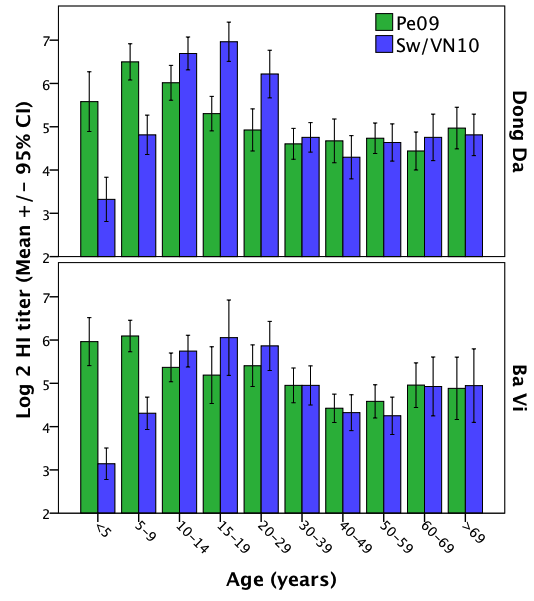


**Supplementary Figure 2.**  Influenza vaccination history among participants with titers to Pe09 and Sw/VN10.


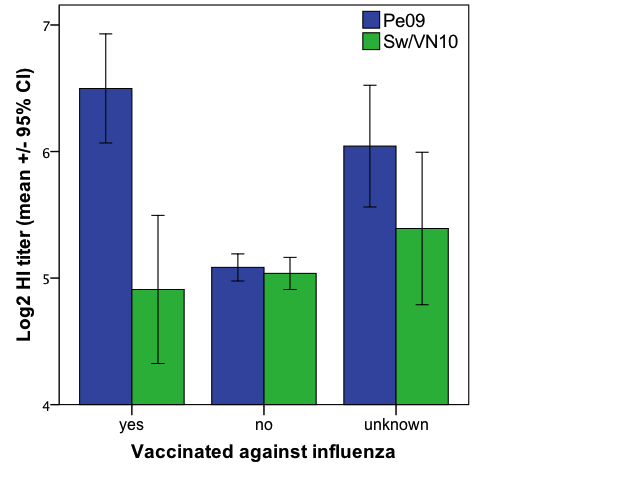


**Supplementary Figure 3**. Profiles of animal contact in urban versus rural settings. (*a*) Direct contact with pigs; (*b*) ownership of domestic animals in households.

**(*a*)**

**(*b*)**
